# Role of the Long Non-Coding RNA *MAPT-AS1* in Regulation of *Microtubule Associated Protein Tau (MAPT)* Expression in Parkinson's Disease

**DOI:** 10.1371/journal.pone.0157924

**Published:** 2016-06-23

**Authors:** Kirsten G. Coupland, Woojin S. Kim, Glenda M. Halliday, Marianne Hallupp, Carol Dobson-Stone, John B. J. Kwok

**Affiliations:** 1 Neuroscience Research Australia, Randwick, NSW, Australia; 2 School of Medical Sciences, University of New South Wales, Sydney, NSW, Australia; UCL Institute of Neurology, UNITED KINGDOM

## Abstract

Studies investigating the pathogenic role of the microtubule associated protein tau (*MAPT*) gene in Parkinson’s disease (PD) have indicated that DNA methylation of the promoter region is aberrant in disease, leading to dysregulated *MAPT* expression. We examined two potential regulators of *MAPT* gene expression in respect to PD, a promoter-associated long non-coding RNA *MAPT-AS1*, and DNA methyltransferases (DNMTs), enzymes responsible for new and maintenance of DNA methylation. We assessed the relationship between expression levels of *MAPT* and the candidate *MAPT-AS1*, *DNMT1*, *DNMT3A* and *DNMT3B* transcripts in four brain regions with varying degrees of cell loss and pathology (putamen, anterior cingulate cortex, visual cortex and cerebellum) in N = 10 PD and N = 10 controls. We found a significant decrease in *MAPT-AS1* expression in PD (p = 7.154 x 10^−6^). The transcript levels of both *MAPT-AS1* (p = 2.569 x 10^−4^) and *DNMT1* (p = 0.001) correlated with those of *MAPT* across the four brain regions, but not with each other. Overexpression of *MAPT-AS1* decreased *MAPT* promoter activity by ∼2.2 to 4.3 fold in an *in vitro* luciferase assay performed in two cell lines (p ≤ 2.678 x 10^−4^). Knock-down expression of *MAPT-AS1* led to a 1.3 to 6.3 fold increase in methylation of the endogenous *MAPT* promoter (p ≤ 0.011) and a 1.2 to 1.5 fold increased expression of the 4-repeat *MAPT* isoform transcript (p ≤ 0.013). In conclusion, *MAPT-AS1* and *DNMT1* have been identified as potential epigenetic regulators of *MAPT* expression in PD across four different brain regions. Our data also suggest that increased *MAPT* expression could be associated with disease state, but not with PD neuropathology severity.

## Introduction

Parkinson’s disease (PD) is a progressive neurodegenerative disorder, characterised by tremor and bradykinesia, that affects 2% of the population over the age of 65 [1]. The neuropathologic hallmarks of PD include loss of dopaminergic neurons in the substantia nigra and the presence of Lewy bodies, cytoplasmic inclusions composed primarily of α-synuclein [2]. Monogenic forms of PD have been linked to mutations in genes including α*-synuclein* (*SNCA*) [3], *leucine-rich repeat kinase 2* (*LRRK2*) [4] and *parkin* (*PARK2*) [5]. These monogenic forms, however, explain only 6% of PD cases, while the remaining are ‘idiopathic’ or sporadic, as their aetiology is unknown [6].

Multiple susceptibility genes have been identified for sporadic forms of PD [7], with *SNCA*, *LRRK2* and the gene encoding microtubule-associated protein tau (*MAPT)* being the most consistently replicated loci [8–10]. Microtubule-associated protein tau (tau), a protein that aids in stabilising the axonal cytoskeleton, is particularly interesting as it has been indicated as a susceptibility gene in other neurodegenerative conditions such as progressive supranuclear palsy [11], and *MAPT* mutations are a cause of monogenic frontotemporal dementia [12]. There are two main *MAPT* haplotypes, termed H1 and H2, resulting from single nucleotide polymorphisms (SNPs) in a region of absolute linkage disequilibrium that spans the entire *MAPT* gene [13]. The major allele, H1, has a higher level of expression than the H2 allele and is associated with increased risk of PD [14]. A recent large-scale Quantitative Trait Loci (QTL) study of brain tissues, comprising N = 773 individuals and measuring transcript levels using the Whole Genome DASL assay, further supported the finding that H1 haplotype elevates gene expression in two brain regions [15]. Given that a higher level of *MAPT* expression may potentially lead to increased neurodegeneration [16] it can be hypothesised that mechanisms regulating the expression of *MAPT* could contribute to neurodegenerative disease. Previously, dysregulation of *MAPT* promoter methylation has been associated with PD [17]. Moreover, in leukocyte DNA, *MAPT* methylation levels also served as a biomarker for age of PD onset in an idiopathic PD cohort [17]. Finally, the *MAPT* promoter has been shown to be aberrantly methylated in both leukocyte and brain tissue of PD patients [17, 18].

**Fig 1:**
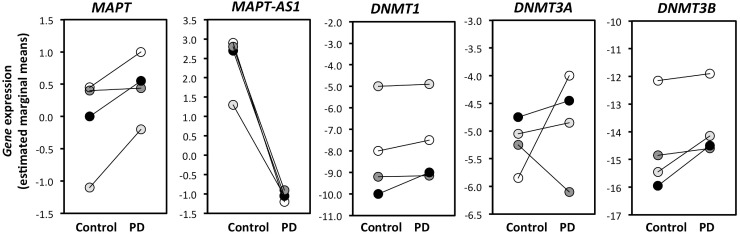
Disease-specific expression levels of *MAPT*, *DNMT1*, *DNMT3A* and *DNMT3B* across four brain regions. Putamen (black circle), ACC (dark grey circle), visual cortex (light grey circle) and cerebellum (open circle). Data points are derived from estimated marginal means after adjusting for significant demographic predictors apart from disease status.

While there are several studies implicating dysregulation of DNA methylation in PD, the mechanisms driving this process are yet to be elucidated. One possible mechanism includes the activity of non-coding RNAs (ncRNAs) which do not code for proteins [19]. One class of ncRNAs are long ncRNAs (lncRNAs) that are diverse in length (≥∼200 bases) and function [20]. An example of the importance of lncRNAs is in the regulation of the neurotrophic and cognition-related gene *BDNF* [21], whose expression is controlled not only by ncRNAs but also by DNA methylation and histone modifications. Of interest, a similar lncRNA exists at the *MAPT* locus: *MAPT-AS1* is an 840 bp lncRNA transcribed from the anti-sense strand of the *MAPT* promoter region [22] (Figure 1 in S1 File). Another mechanism includes DNA methyltransferases (DNMTs) which are enzymes carrying a C-terminal catalytic domain that methylates CpG dinucleotides. Catalytically active members of the group include Dnmt1 (gene: *DNMT1*), responsible for maintenance of methylation by targeting hemimethylated DNA, and Dnmt3a (gene: *DNMT3A*) and Dnmt3b (gene: *DNMT3B*), which catalyse *de novo* methylation and are mainly active during embryonic development and neurogenesis in brain tissue [23]. Aberrant expression of *DNMT3A* has been linked to atypical DNA methylation levels in frontotemporal dementia [24]. Similarly, alterations to the activity of Dnmt3a and Dnmt1 have been implicated in neuronal cell death pathways in mice and amyotrophic lateral sclerosis in humans [25].

We postulate that dysregulation of *MAPT* expression in PD is due to altered levels of the lncRNA *MAPT-AS1* and/or at least one *DNMT* gene in a disease-specific manner. In this study we examined the relationship between *MAPT*, *MAPT-AS1*, *DNMT1*, *DNMT3A* and *DNMT3B* expression levels in PD and control brain tissue samples across four brain regions. We found disease-specific effects of *MAPT-AS1* and *DNMT1* on *MAPT* expression. *In vitro* cellular assays established that *MAPT* promoter activity is altered via an epigenetic mechanism when *MAPT-AS1* expression is manipulated *in vitro*.

## Materials and Methods

### Study cohorts

We examined 80 brain tissue samples from 10 PD and 10 age- and sex-matched controls (Table 1) across four brain regions to assess underlying disease-specific effects that are independent of differences in pathology and cell loss: putamen (affected early in PD and with tissue loss), anterior cingulate cortex (ACC, affected later in PD and with minimal cell loss), visual cortex (VC, no overt pathology but with abnormal lipid metabolism [26]) and cerebellum (not affected by PD) [27, 28]. All PD cases were Braak stage IV or higher. All cases with head injury, brain tumour, infarction or other significant neurodegenerative pathologies were excluded. Controls had no history of neurological or psychiatric symptoms and no significant neuropathology. These samples were all of Caucasian descent and were provided by the Sydney Brain Bank (HC15008 –ethics approval provided by the University of New South Wales Human Research Ethics Committee) and the NSW Tissue Resource Centre (Protocol No X15-0199 & HREC/11/RPAH/147 –ethics approval provided by the NSW Government Health Sydney Local Health District). Ethics approval for the analyses of human tissue for epigenetic modifications (HREC 11312) was provided by the University of New South Wales Human Research Ethics Committee). Informed written consents were obtained from all participants, or their next of kin, or legal guardian, as the participants were recruited, using procedures approved by the ethic committees detailed above.

**Table 1:** Comparison of demographics and *MAPT* haplotype frequency in brain tissue cohort.

Disease status		Sex		Age(y)^a^		*MAPT* haplotype		PMD (h)^a^	Tissue pH^a^
	N	M	F	Mean ± SD	Range	H1	H2	Mean ± SD	Mean ± SD
Normal	10	7 (70%)	3 (30%)	77.4 ± 8	65–88	7 (70%)	3 (30%)	21.3 ± 15	6.5 ± 0.2
PD	10	7 (70%)	3 (30%)	79.5 ± 6	69–90	7 (70%)	3 (30%)	19.4 ± 12.9	6.5 ± 0.7

^a^ There were no significant differences in mean age at death, tissue pH and post-mortem delay (PMD) between cases and controls as assessed by Student’s t-test.

F, female; M, male; *MAPT*, microtubule-associated protein tau; PD, Parkinson’s disease; PMD, post-mortem delay; SD, standard deviation.

### Nucleic acids extraction and genotyping

Genomic DNA and RNA were extracted from brain tissue using DNeasy and RNAQuick kits (QIAGEN, Venlo, Netherlands). A Taqman Probe Genotyping Assay (Life Technologies, CA, USA) for rs1052553 was used to determine the corresponding H1/H2 *MAPT* diplotype in brain tissue.

### Haplotype-specific methylation analysis

Genomic DNA (gDNA) samples (500 ng) were bisulfite converted using the EpiTect96 Bisulfite Kit (QIAGEN, Venlo, Netherlands) according to manufacturer’s specifications. PCR primers were designed from UCSC Feb 2009 (hg19) draft to amplify a 148 bp region of the *MAPT* promoter region in bisulfite-converted gDNA (GRCh37_Chr 17: 43,971,386–43,971,534). PCR and sequencing primer design was performed using ABI Methylprimer v1.0 (ABI) and PyroMark Assay Design v2.0 software (QIAGEN). Haplotype-specific pyrosequencing was based on the design of haplotype-specific primers which have either a ‘G’ or ‘A’ nucleotide at its 3’ end, corresponding to bisulfite converted sequence of either the H1 or H2 allele of the rs76594404 polymorphism within the PCR amplified region (Figure 1 in S1 File) as described previously [17]. Bisulfite converted DNAs were amplified using MAPT1_pyroF_biotin 5’-GGGAAAGAGATTTTAGTTAGG-3’, MAPT1_pyroR 5’-CCCTTTACTTTCAATCAA-3’, and sequenced using primers specific to either the H1 (5’-gtaaactaccttccacttaac-3’) or H2 (5’-gtaaactaccttccacttaaa-3’) haplotypes (Figure 1 in S1 File). The CpG dinucleotides at position 2 to 6 in the pyrosequenced region showed high intra- and inter- experiment correlation of methylation levels and thus the arithmetic mean of these CpGs was used as a summary score for our analyses.

### Relative quantification of gene expression

Approximately 100mg of frozen tissue, from each brain region of each patient, was extracted using the RNAeasy Mini Kit (Qiagen, Valencia, CA, USA). SuperScript III First Strand cDNA synthesis kit (Life Technologies, CA, USA) was used to generate cDNA from brain total mRNA (100 ng) as directed by manufacturer’s instructions. Transcript levels for *MAPT* (either total using primers that span exons 11 and 12, or 4-repeat *MAPT* isoform using primers that span exons 9 and 11) and *MAPT-AS1* were assayed using relative quantification–polymerase chain reaction (RQ-PCR). Two housekeeping genes, *succinate dehydrogenase complex*, *subunit A* (*SDHA*) [29] and the *eukaryotic translation initiation factor 4A2* (*EIF4A2*) [30] were used to normalize gene expression levels. Primers for the validation of each gene were designed to span exon/exon boundaries to minimise the effects of genomic DNA contamination. The complete set of primers is listed in Table 1 of S1 File. Four experimental replicate PCR reactions were performed for each sample using 1nM concentration of each primer in 1x Platinum SYBR Green qPCR Supermix–UPG [Invitrogen, Carlsbad, CA, USA) and 1ng of reverse transcribed RNA. Serial dilutions of template cDNAs confirmed that each primer pair had amplification efficiency of ∼2. Transcript levels of the *DNMT* genes were assayed using commercially available pre-designed Taqman Probes [*DNMT1* [Hs00945875_m1), *DNMT3A* [Hs01027166_m1) and *DNMT3B* [Hs00171876_m1) [Life Technologies)). Four experimental replicate qPCR reactions were performed for each Taqman probe in 1x Taqman Fast Advanced Master Mix [Life Technologies) with 1ng of cDNA. Gene expression levels [2^-∆Ct^ where -∆Ct = —Ct [target)–Ct [geometric mean of house-keeping genes) [29, 31]) were log transformed to -∆Ct values to improve linearity of data in the Repeat Measure Mixed Linear Model Regression analyses, or expressed as 2^-∆∆Ct^ where ∆∆*Ct* = (*Ct*(*target gene*, *control cells*)−*Ct*(geometric mean of house-keeping genes, *control cells*))−(*Ct*(*target gene*, *treated cells*)−*Ct*(geometric mean of house-keeping genes, *treated cells*)) when we were comparing the difference in means between control and treated cells within each independent transfection.

### *MAPT-AS1* expression constructs and siRNA

A full length 840bp cDNA corresponding to the *Homo sapiens* MAPT antisense RNA 1 (*MAPT-AS1*) non coding RNA transcript sequence (NR_024559.1) was commercially synthesized and subcloned into the expression vector pCDNA3.1(+) (GenScript, NJ, USA). Knock-down expression of *MAPT-AS1* was achieved using a Custom Silencer® Select siRNA (s237111) (Ambion–Thermofisher Scientific, NY, USA). The sequence of the s237111 siRNA is: Sense 5’-CCACUUCAUGGAUAAGUAAtt-3’ and Antisense 5’-UUACUUAUCCAUGAAGUGGgt-3’. The Silencer® Negative Control No. 2 siRNA (Ambion–Thermofisher Scientific) served as a negative control for non-specific effects.

### Cellular assays

The *MAPT*-promoter luciferase vector, *MAPT*-pCpG, was as described previously [17]. Two cell lines, the neuroblastoma SK-N-MC (ATTC HTB-10) and the human embryonic kidney HEK293 (ATCC CRL-1573) were co-transfected with either the *MAPT-AS1* cDNA expression construct, or siRNAs, and the *MAPT*-pCpG constructs using Lipofectamine 2000 (Invitrogen, CA, USA). 1.5 x 10^4^ cells/well in a 24 well plate (Nunc Delta Si, Thermofisher Scientific, NY, USA) were transfected with 2μl Lipofectamine 2000, 200ng cDNA expression construct or 2pmol siRNA, 200ng *MAPT*-pCpG luciferase constructs. An additional 20ng of the pRL-TK renilla luciferase vector (Promega, WI, USA) was added to the transfection mixture for the normalization of transfection efficiencies. We examined whether there was a haplotype-specific effect on promoter activity by co-transfecting *MAPT*-promoter constructs corresponding to either the H1 or H2 haplotype. Cells were lysed after 48 h and assayed for luciferase activity using the Dual-Luciferase® Reporter assay system (Promega). Results were obtained from 5 independent experiments. No significant differences in raw renilla luciferase values were observed between experimental groups (data not shown).

### Statistical analyses

Repeat Measure Mixed Linear Model Regression analysis, with a random intercept model, was used to explore the relationship between transcript levels and disease status across four brain regions. Graphical outputs for these analyses were generated using the alternate form of the repeat measure linear regression model syntax, known as general linear model. Gene expression levels are presented as estimated marginal means because they are adjusted for the effects of significant covariates. The syntax for the statistical analyses are given in full in the Methods of S1 File. Of the cohort demographics (apart from disease status), tissue pH was the only significant predictor for *MAPT* transcript levels. Thus, relationships between gene expression levels of *MAPT*, *MAPT-AS1*, *DNMT1*, *DNMT3A* and *DNMT3B*, were assessed with tissue pH as covariate. Significance of results was adjusted for multiple testing using the Bonferroni correction. Student’s paired samples t-tests were used to assess *in vitro* promoter luciferase activity, changes in endogenous DNA methylation levels and transcript levels from cell culture experiments. All statistical analyses were performed in SPSS v. 22 (IBM, Armonk, NY).

## Results and Discussion

### Disease-specific differences in gene expression levels

Student’s independent samples *t*-test was used to examine age, tissue pH, and post-mortem delay between groups, finding no significant difference between PD cases and controls (Table 1). Gender and *MAPT* haplotype have been previously demonstrated to significantly affect *MAPT* methylation [17], as such our controls and PD tissue samples were gender-matched and had identical *MAPT* haplotype frequencies. In addition to the basic demographic factors such as age (Table 2), we specifically examined the effect of disease state on the transcript levels of our five genes of interest, *MAPT*, *MAPT-AS1*, *DNMT1*, *DNMT3A* and *DNMT3B*. The Repeat Measure Mixed Linear Model Regression analysis is designed to detect consistent changes across the four brain regions that differ in their level of pathology and cell loss. We speculate that consistent changes in gene expression across four brain regions are more likely to represent factors that are causative of disease, rather than a secondary phenomenon of disease progression. As shown in Table 2, univariate analyses identified that disease state was the strongest predictor for *MAPT-AS1* transcript levels (t = -6.287, p = 7.154 x 10^−6^), followed by *DNMT3B* (t = 4.044, p = 7.977 x 10^−5^) and *DNMT3A* (t = 3.005, p = 0.007). Graphical representations of the change in gene expression with disease state for the five genes are shown in Fig 1.

**Table 2:** Univariate Repeat Measure Mixed Linear Model Regression analyses for predictors of gene expression.

* *	*MAPT*	* *	*MAPT-AS1*	* *	*DNMT1*	* *	*DNMT3A*	* *	*DNMT3B*	* *
Predictor	t-Statistic	p value^a^	t-Statistic	p value	t-Statistic	p value	t-Statistic	p value	t-Statistic	p value
**Age**	0.87	0.449	-0.0776	0.447	1.821	0.085	1.02	0.32	-0.152	0.88
**Gender**	0.811	0.474	-0.298	0.769	-0.563	0.597	-0.378	0.709	2.271	**0.042**
**PMI**	-0.504	0.655	0.103	0.919	-0.385	0.718	-0.551	0.588	0.32	0.753
**Tissue pH**	-3.212	0.004	-0.594	0.559	1.022	0.378	-2.039	0.057	-0.048	0.963
**Disease status**	1.551	0.175	-6.287	**7.15E-05**	2.262	0.396	3.005	0.007	4.044	**7.98E-05**

^**a**^ significant p values (p < 0.05) are highlighted in bold. Note that the significance levels are not adjusted for multiple testing as this is an exploratory analysis.

### Predictors of *MAPT* expression levels

We performed multivariate analyses to explore the relationships between different gene transcript levels and the effect of disease status. We set the threshold p value at < 0.004 for 12 comparisons (4 genes x 3 variables of disease, gene transcript levels, and disease * gene transcript levels). As shown in Table 3, *MAPT-AS1* transcript levels was the best predictor of *MAPT* transcript levels (t = 5.656, p = 2.569 x 10^−4^) with a significant interaction with disease state (t = -4.286, p = 0.001). *DNMT1* transcript levels were also significantly associated with *MAPT* transcript levels (t = 4.476, p = 0.001), but without significant interaction with disease status after correction for multiple testing. Of interest, *DNMT1* transcript levels were not associated with *MAPT-AS1* levels (Table 3), suggesting independent effects on *MAPT* expression. Neither *DNMT3A* nor *DNMT3B* were significantly associated with *MAPT* or *MAPT-AS1* transcript levels (Table 3).

**Table 3:** Multivariate Repeat Measure Mixed Linear Model Regression analyses for the effects of disease status and *MAPT* transcript levels.

	*MAPT*		*MAPT-AS1*	
Predictor^a^	t-Statistic	p value^b^	t-Statistic	p value^b^
**Disease**	-1.456	0.179	-	-
***MAPT1-AS1***	5.656	**2.57E-04**	-	-
Disease * *MAPT-AS1*	-4.286	**0.001**	-	-
**Disease**	-2.365	0.027	0.825	0.415
***DNMT1***	4.476	**0.001**	0.849	0.407
Disease * *DNMT1*	-2.991	0.006	-1.014	0.318
**Disease**	-0.551	0.583	0.696	0.49
***DNMT3A***	1.512	0.135	2.62	0.013
Disease * *DNMT3A*	-0.941	0.35	-0.875	0.386
**Disease**	-1.121	0.311	-0.201	0.841
***DNMT3B***	-2.407	0.062	0.066	0.948
Disease * *DNMT3B*	-0.791	0.463	-0.995	0.302

^a^ For each gene of interest, demographic covariates were included in the multivariate regression analyses if they were significantly associated in the initial univariate analysis (Table 2). *For MAPT and MAPT-AS1*: *Tissue pH; MAPT and DNMT1*: *Tissue pH; MAPT and DNMT3A*: *Tissue PH; MAPT and DNMT3B*: *Tissue pH and Gender*. *For MAPT-AS1 and DNMT3B*: *Gender*.

^b^ p values that survived correction for multiple testing are indicated in bold.

### Effect of *MAPT-AS1* expression on the activity of a *MAPT* promoter construct

**Fig 2:**
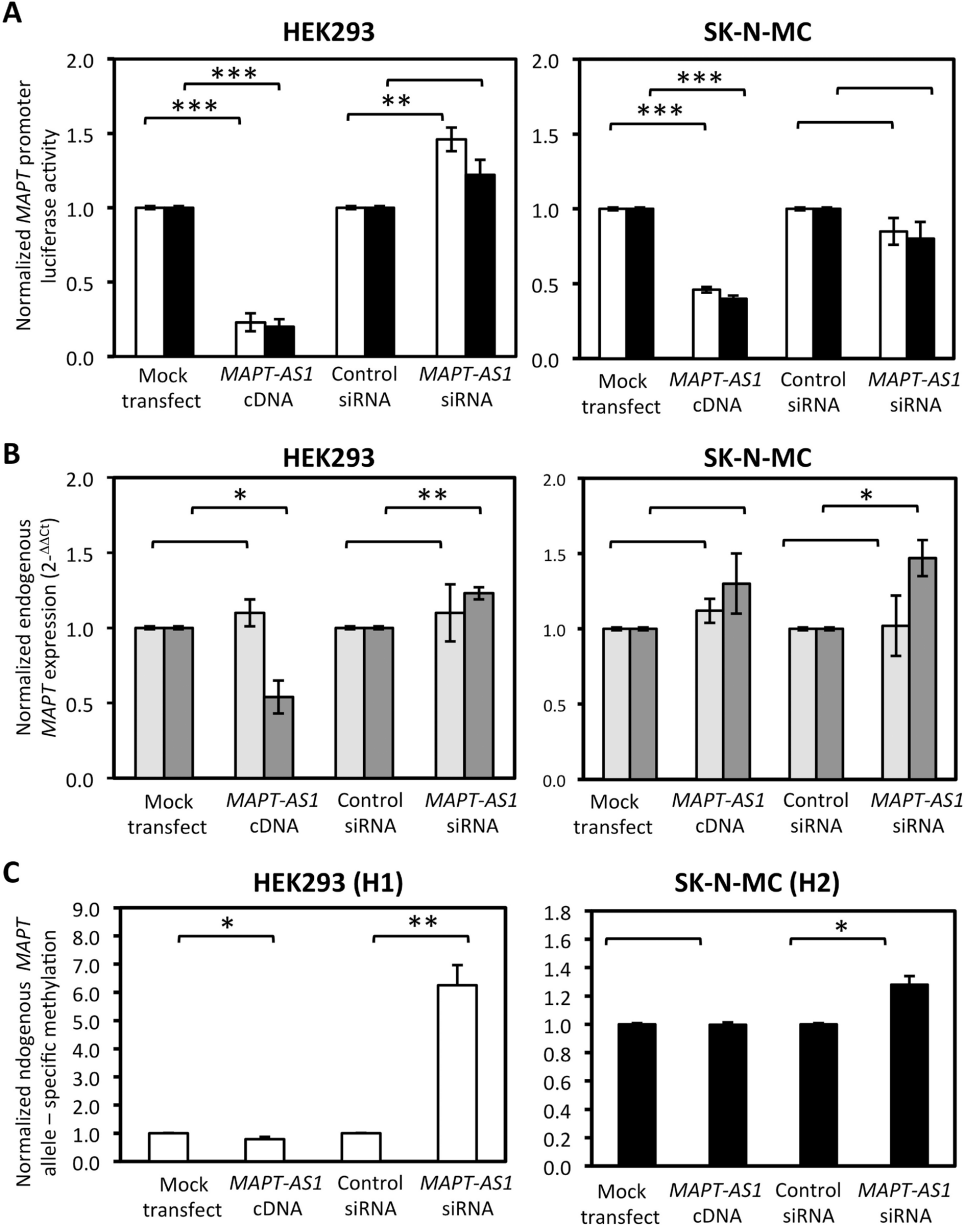
Effect of *MAPT-AS1* over- and knock-down expression on *MAPT* expression in HEK293 and SK-N-MC cells. A) H1 (white columns) and H2 (black columns) haplotype *MAPT* promoter-driven luciferase activity. Luciferase activity is normalized to each control transfection levels. B) Endogenous transcript levels of either total *MAPT* (light grey columns) or 4 repeat *MAPT* transcript (dark grey columns). Transcript levels are normalized to each control transfection levels C) Endogenous haplotype-specific DNA methylation with H1 (white columns) and H2 (black columns) specific data indicated. Methylation levels from *MAPT-AS1* over- or under-expression are normalized to control transfection levels. Error bars indicate standard error of the mean from 5 independent experiments. *, p < 0.05; *** p < 0.0001.

As *MAPT-AS1* expression was significantly correlated with *MAPT* expression, we identified a causative relationship between the two loci *in vitro* using a sensitive reporter assay in two model cell lines. We co-expressed the *MAPT-AS1* cDNA expression construct with *MAPT* promoter luciferase constructs corresponding to either the H1 or H2 haplotype. In HEK293 cells, for both *MAPT* haplotypes, there was a significant decrease in luciferase activity when the lncRNA was overexpressed (H1 haplotype, 4.3 fold decrease, p = 2.678 x 10^−4^; H2 haplotype, 5.1 fold decrease, p = 9.203 x 10^−5^). A similar effect was observed for SK-N-MC cells (H1 haplotype, 2.2 fold decrease, p = 9.159 x 10^−6^; H2 haplotype, 2.5 fold decrease, p = 9.855 x 10^−6^) when compared to mock transfected cells (Fig 2A). Conversely, when the endogenous level of *MAPT-AS1* was knocked-down by siRNA specific to the lncRNA, we observed a significant increase in activity from the H1 haplotype reporter construct in HEK293 cells (1.5 fold increase in luciferase activity, p = 0.004), but not the H2 haplotype. No significant effects were observed for either haplotypes in SK-N-MC cells (Fig 2A).

### *MAPT-AS1* expression and effects on endogenous *MAPT* expression

In order to determine whether increased *MAPT-AS1* expression influenced *MAPT* expression via *MAPT* promoter methylation we examined endogenous *MAPT* methylation and expression *in vitro* using the same cellular models as above, but omitting the co-transfection of the *MAPT* promoter luciferase constructs. For these experiments, we measured *MAPT-AS1* transcript levels in transfected cells by RQ-PCR, and confirmed that transfection with *MAPT-AS1* cDNA, compared to mock transfected cells, led to a significant 573- to 2227-fold increase in gene expression for SK-N-MC (p = 0.002) and HEK293 (p = 0.013) cells respectively (Figure 2A in S1 File). Similarly, transfection with the *MAPT-AS1* siRNA, compared with the control siRNA, led to a significant 1.7-fold knock-down (or 60% of pre-knock-down levels) of endogenous *MAPT-AS1* transcript levels in both SK-N-MC (p = 0.042) and HEK293 cells (p = 0.045) (Figure 2B in S1 File). We also noted that there were no significant differences in the levels of our two house-keeping genes *SDHA* and *EIF4A2* between any experimental groups (data not shown). For endogenous *MAPT* expression, we measured both total transcript levels as well as the levels of the longer isoform with an additional alternatively spliced exon (4 repeat *MAPT* isoform). In HEK293 cells, we observed a significant 1.9 fold decrease in transcript levels for the 4 repeat *MAPT* isoform (p = 0.015), but not for total *MAPT* transcripts (Fig 2B), in cells over-expressing the *MAPT-AS1* cDNA compared with mock transfected cells. No significant effect of *MAPT-AS1* over-expression on endogenous *MAPT* transcript levels was observed in SK-N-MC cells (Fig 2B). Conversely, when we knocked-down expression of *MAPT-AS1*, we observed a significant 1.2- to 1.5-fold increase in 4 repeat *MAPT* transcripts for HEK293 cells (p = 0.005) and SK-N-MC cells (p = 0.019) respectively, compared to cells transfected with the control siRNA.

### *MAPT-AS1* expression and effects on endogenous *MAPT* promoter methylation

HEK293 and SK-N-MC cells differ in their *MAPT* haplotypes with HEK293 reported to be H1/H1 homozygotes and SK-N-MC cells reported to be H1/H2 heterozygotes [32]. We performed haplotype-specific pyrosequencing to determine the haplotype-specific effects of *MAPT-AS1* expression on DNA methylation of the *MAPT* promoter. In HEK293 cells, we observed a 1.4 fold decrease in DNA methylation (p = 0.032) of the H1 haplotype when we over-expressed the *MAPT-AS1* cDNA compared to mock-transfected cells (Fig 2C). Conversely, we observed a marked 6.3 fold increase in DNA methylation (p = 0.002) on the H1 haplotype when we knocked-down the expression of the lncRNA (Fig 2C). In the SK-N-MC cells, the assay for the H1 haplotype failed. Sanger sequencing across the pyrosequencing primer binding sites from both cell lines confirmed the presence of the appropriate homozygote genotype of rs76594404 in HEK293 and heterozygote genotype for SK-N-MC cells (Figure 3 in S1 File). However, the sequencing also revealed that there was higher level of basal DNA methylation in two CpG sites within the primer binding site for SK-N-MC cells compared with HEK293 cells (Figure 3 in S1 File). This could have affected the binding of the H1 haplotype-specific sequencing primer in SK-N-MC cells. For the H2 haplotype in SK-N-MC cells, we observed a 1.3 fold increase in DNA methylation (p = 0.011) when we knocked-down the expression of the lncRNA.

In this study we examined the expression of potential epigenetic regulators of gene expression, the lncRNA *MAPT-AS1* and the methyltransferase genes *DNMT1*, *DNMT3A* and *DNMT3B*, and their relationship to *MAPT* expression across four brain regions with differing PD pathology. Of note, *MAPT-AS1* expression was highly associated with disease state, and was the strongest predictor of *MAPT* expression of the four candidates genes. Over-expression of *MAPT-AS1 in vitro* resulted in decreased *MAPT* expression in two model cell lines, whilst knock-down expression of the lncRNA had the opposite effect, most clearly observed on the methylation and expression from the endogenous *MAPT* promoter.

The four brain regions included in this study, the putamen, anterior cingulate and visual cortices and cerebellum, differ in their level of PD neuropathology, as defined by α-synuclein deposition as well as tissue loss. We report for the first time a significant PD-specific decrease in *MAPT-AS1* expression across these four brain regions, suggesting that this decrease occurs independently of alpha-synuclein deposition and cell loss. Certain promoter-associated lncRNAs, such as the antisense *BDNF-AS*, have been shown to act as a repressor of gene expression via epigenetic modifications. This was demonstrated in mice where inhibition of the endogenous *Bdnf-AS* results in upregulation of the mouse *Bdnf* expression, changes in histone modifications and increased neuronal growth and differentiation [21]. We observed a similar inhibitory effect on *MAPT* expression by *MAPT-AS1*. Future studies will confirm whether the effect of the lncRNA is specific for the *MAPT* locus, or whether it has other trans-acting effects as well. The role of these promoter-associated lncRNAs are of special interest as we have noted that other neurodegenerative genes, such as the alpha-synuclein (*SNCA*) [3] and glycogen-synthase kinase 3beta (*GSK3B*) genes [33], also have similar lncRNAs (*SNCA*-*AS* and *BC035247* respectively).

*MAPT* haplotypes are major determinants of *MAPT* gene expression as well as disease risk [7, 15, 34, 35]. Of note, there are studies that support the role of the H1/H2 haplotypes on differential splicing [32, 36] rather than elevation of total gene expression. Current QTL studies that use gene expression arrays do not effectively capture all alternative splicing information, and there are potential artifacts that can arise from polymorphisms that fall within the binding regions of the probes. Future studies from next generation transcriptomics analyses, which do not rely on hybridization of probes, will provide more accurate information of the expression levels of all splice variants and should provide conclusive evidence on this matter.

Recent studies have suggested that gene transcription and the regulation of alternative splicing are functionally linked [37] and may be due to recruitment of splicing factors by RNAPII, particularly SR proteins such as SRp20 which have been shown to modulate alternative splicing of *MAPT* [32]. Moreover, epigenetic modifications including DNA methylation also play a crucial role in regulating alternative splicing and have implications for disease process [38]. A DNA-binding protein, CCCTC-binding factor (CTCF), can promote inclusion of alternatively spliced exons by mediating local pausing of RNA polymerase II, and CTCF binding to these exons is inhibited by DNA methylation [39]. In brain tissue of patients with autistic spectrum disorders, altered expression and alternative splicing of *SHANK3* gene were observed in samples with increased methylation of CpG islands within *SHANK3*. Furthermore, a DNA methylation inhibitor modified the methylation of these CpG islands and altered the isoform-specific expression of *SHANK3* in cultured cells [40].

Promoter-associated lncRNAs, such as *MAPT-AS1*, can impact on gene expression *in trans*, often by direct interaction with key regulators of DNA methylation. Di Ruscio *et al*. showed that a lncRNA arising from the *CEBPA* gene locus, termed extra-coding *CEBPA*, was required for regulation of DNA methylation at this site through the association of the lncRNA with DNMT1 [41]. Similarly, the sustained expression of a central nervous system expressed intergenic lncRNA, *Dali*, was required for the differentiation of neuroblastoma cells via interactions with DNMT1 and regulates DNA methylation status of multiple CpG island-associated promoters *in trans* [42]. In our cellular assays, we confirm that the elevation in DNA methylation (in our pyrosequenced region) was significantly associated with an increase in the alternatively spliced 4-repeat *MAPT* isoform levels, but not total *MAPT* transcripts. This provides further evidence that epigenetic modifications play a role in determining both gene transcription and alternative splicing.

Certain neuroprotective compounds, such as vitamin E, have been shown to have a haplotype-specific effect on *MAPT* expression via epigenetic mechanisms as well [17]. Therefore, it was important to demonstrate that *MAPT-AS1* was capable of inhibiting the expression of both the H1 and H2 promoter luciferase reporter constructs. We observed a significant effect on each haplotype as assayed in the two cell lines used in this study. The luciferase reporter assays also suggested that *MAPT-AS1* can act to repress gene expression *in trans*.

We noted cell type-specific differences in our two cellular models. Firstly, in control HEK293 cells, endogenous *MAPT-AS1* was expressed approximately 30-fold higher than endogenous *MAPT* (data not shown), whilst in control SK-N-MC cells, *MAPT-AS1* was expressed approximately 1.1-fold lower than *MAPT* (data not shown). Secondly, transfection of the *MAPT-AS1* cDNA resulted in a higher level (11-fold) of expression in the HEK cells compared with SK-N-MC cells (Figure 2 in S1 File). The relatively low level of expression of the transfected transgene or transfection efficiency in neuronal cell types has been observed previously [43]. At present, it is unclear whether these differences could fully explain the more consistent and significant effects on luciferase activity, endogenous gene expression and DNA methylation in HEK293 cells compared with SK-N-MC cells. Another aspect we should consider is the difference between over-expression and knock-down expression of *MAPT-AS1* and their corresponding effects on the *MAPT*-promoter luciferase activity (Fig 2A). In the over-expression assays, we observed a consistent effect in both cell lines as we increased the copy numbers of the lncRNA and its putative target on the *MAPT* promoter by our transfections. Conversely, in the knock-down assays, we only increased the copy number of the exogenous *MAPT* promoter, whilst knocking down the endogenous lncRNA. This may have led to failure to detect a consistent effect of siRNA knock-down on the luciferase assays.

Finally, we also examined other candidate trans-acting factors of *MAPT* expression, specifically the expression profiles of the three *DNMT*s. In terms of *MAPT* expression, only *DNMT1* transcript levels were significantly associated across the four brain regions, although there does not appear to be a disease-specific effect after adjusting for multiple testing. *DNMT1* is required for maintenance of methylation, gene regulation and chromatin stability. *DNMT1* is of interest as mutations in this gene have been associated with hereditary sensory neuropathy with dementia and hearing loss [44]. Both *DNMT3A* and *DNMT3B* levels were significantly associated with disease state but not with *MAPT* levels in our cohort of brain samples. This is in contrast to a study by Iwata *et al*. [45] which demonstrated that *DNMT3A* over-expression was capable of inhibiting *MAPT* expression in a cellular model.

## Conclusions

The main limitation of our study was the relatively small sample size. Future studies with a larger number of brain samples may also resolve apparent contradictions between tissue and cellular studies for the *DNMT3A* gene. Moreover, while our pyrosequencing assay focused on 5 CpGs within the CpG island of the *MAPT* promoter, the examination of additional CpGs in non-proximal promoter regions may identify regions whose methylation levels more accurately reflect *MAPT-AS1-*mediated *MAPT* expression changes. In conclusion, we have identified a lncRNA which has an inhibitory effect on the expression of a major neurodegenerative gene, *MAPT*. The lncRNA may serve as a sensitive biomarker of disease state in PD, as well as a potential therapeutic target for inhibiting the expression of *MAPT*.

## Supporting Information

S1 File Supporting File. (PDF)
