# Supplementary material for: Role of the Long Non-Coding RNA MAPT-AS1 in Regulation of Microtubule Associated Protein Tau (MAPT) Expression in Parkinson's Disease
Source: PLoS One. 2016 Jun 23;11(6):e0157924. doi: 10.1371/journal.pone.0157924 (PMC4919105; doi:10.1371/journal.pone.0157924)
Supplement: S1 File — (PDF) [file pone.0157924.s001.pdf]

Table 1. PCR primers for validating changes in gene expression by semi-quantitat RT-PCR

Gene Name	Primer Name	Sequence	Predicted size (bp)
MAPT	MAPT-4repeat-RTF	AGCAACGTCCAGTCCAAGTG	150
MAPT	MAPT-4repeat-RTR	ACCTCCTGGTTTATGATGGATG	
MAPT	MAPT-total-RTF	TGTGGCTCATTAGGCAACAT	91
MAPT	MAPT-total-RTR	ACTGGACTCTGTCCTTGAAGTC	
MAPT-AS1	MAPT-AS1-RTF	AGATGCACCTGCAGCCC	121
MAPT-AS1	MAPT-AS1-RTR	CCCGTCCTTGTTCTGACTCC	
EIDF4A2	EIDF4A2-RTF	GGTCAGGGTCAAGTCGTGTT	135
EIDF4A2	EIDF4A2-RTR	CCCCTCTGCCAATTCTGTGA	
SDHA	SDHA-RTF	TGGGAACAAGAGGGCATCTG	86
SDHA	SDHA-RTR	CCACCACTGCATCAAATTCATG	

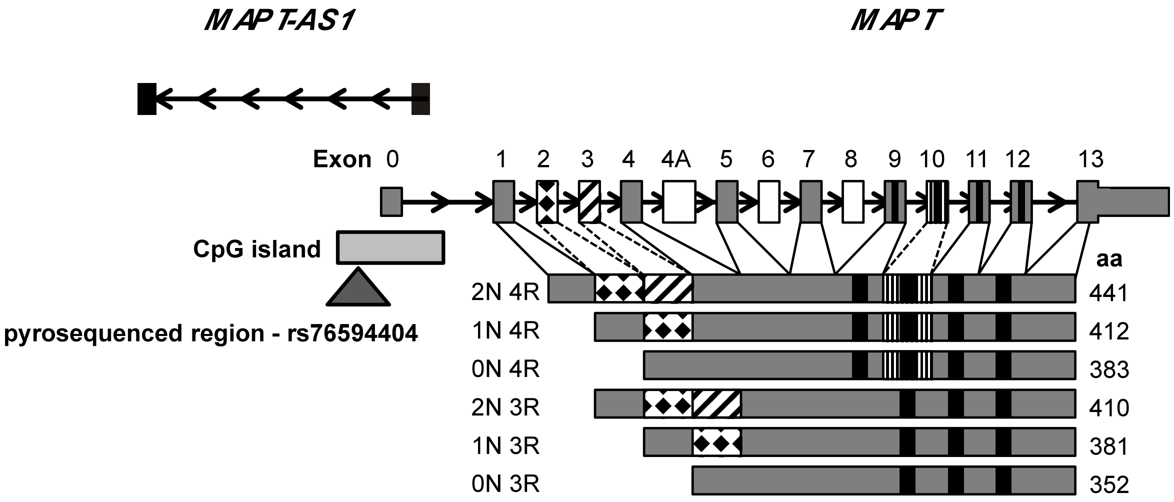

Figure 1. Schematic diagram of the *MAPT-AS1* and *MAPT* genes (not drawn to scale) and protein variants arising from alternative splicing. Relative orientations of the genes are indicated by arrows. The *MAPT* CpG island (light grey box) and the position of the H1/H2 *MAPT* haplotype tagging polymorphism (rs765994404) are also indicated. Alternatively spliced exons are indicated by dashed lines. Schematic of *MAPT* exons with their traditional notation, denoting exons present in all isoforms (grey), exons that are not present in adult central nervous system splice isoforms (white), and alternatively spliced exons 2 (checked), 3 (striped) & 10 (dotted). Microtubule-binding domains are depicted as vertical black bars. The six isoforms present in human brain are depicted below. Alternative splicing of exons 2 and 3 results in proteins with 0, 1 or 2 N-terminal inserts (ON, 1N, 2N), while exon 10 alternative splicing generates proteins with either 3 or 4 microtubule-binding repeats (3R or 4R).

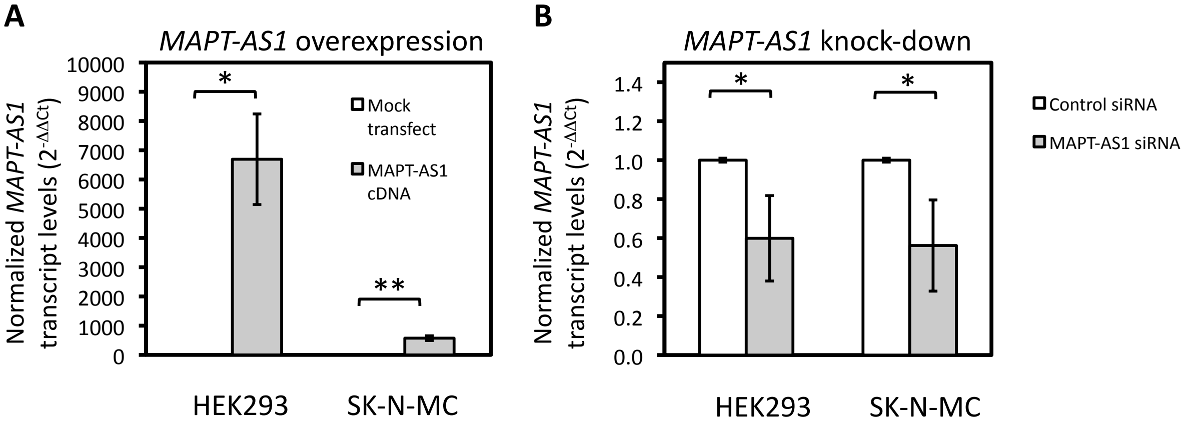

Figure 2. Relative changes in levels of *MAPT-AS1* transcripts ($2^{-\Delta\Delta Ct}$) in HEK293 and SK-N-MC cells after (A) overexpression (grey columns) or (B) knock-down expression (grey columns) compared with control treatments (open columns). Note that for each independent experiment, the expression level of *MAPT-AS1* in the control cells is normalized to '1' and the change in *MAPT-AS1* in the treated cells adjusted accordingly. Error bars indicate standard error of the mean from 5 independent experiments. *, $p < 0.05$; ** $p < 0.005$.

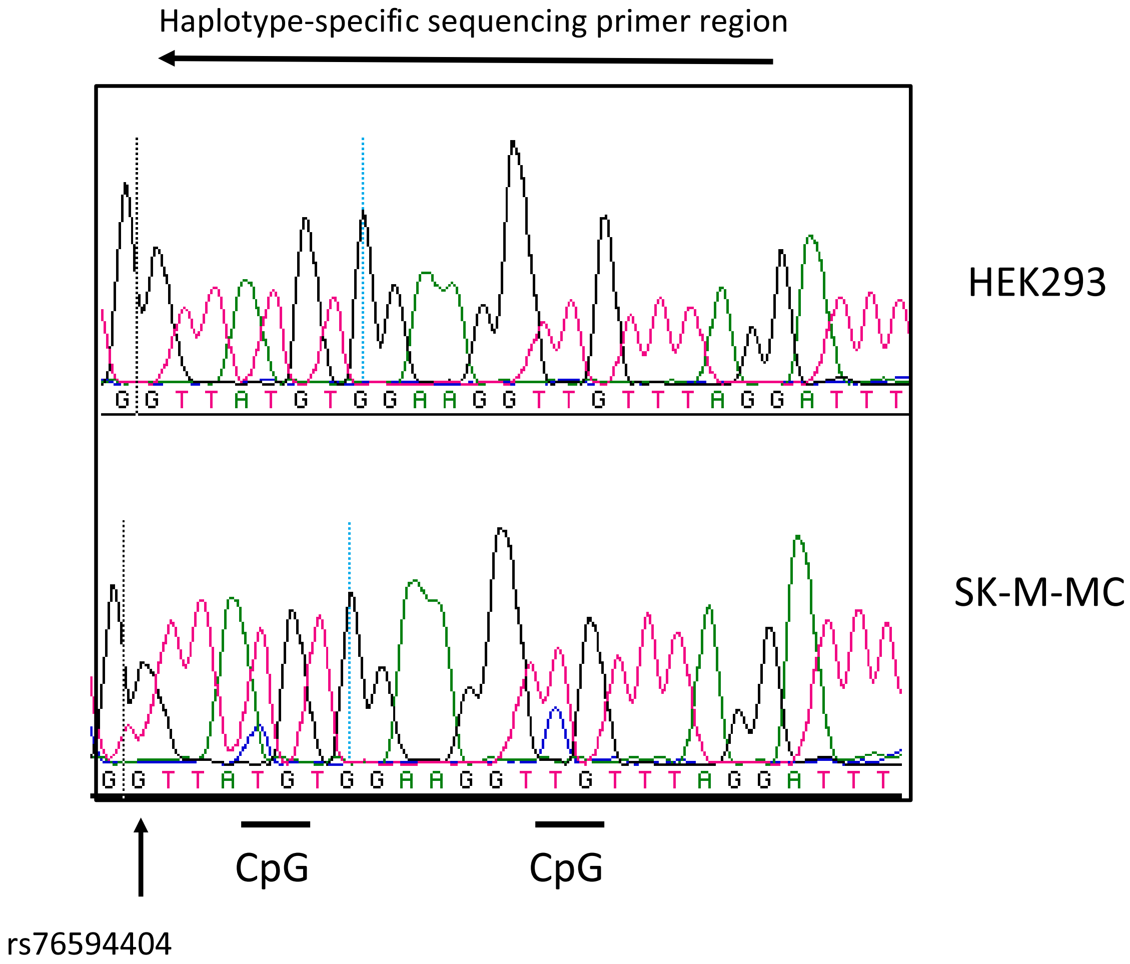

Figure 3. Electropherogram traces from Sanger sequencing of PCR products arising from amplification of bisulfite converted DNAs. The sequence data covers the primer binding site of the primer used to generate haplotype-specific DNA methylation data. Note the appropriate GT heterozygote genotype for rs76594404 for SK-N-MC cells. Also note the relatively high level of DNA methylation in SK-N-MC compared with HEK293 cells, as indicated by the height of the ‘C’ peaks at the two CpG sites.

Methods

SSPS Syntax 1 – To examine the relationship between dependent variable [-ΔCt values of gene of interest] and independent variable [demographic data]. Repeat measure of gene expression are for the different brain regions [Brain_regions] in each patient [PatientID].

MIXED [-ΔCt value of gene of interest] WITH [demographic data]

/CRITERIA=CIN(95) MXITER(100) MXSTEP(10) SCORING(1) SINGULAR(0.000000000001) HCONVERGE(0, ABSOLUTE) LCONVERGE(0, ABSOLUTE) PCONVERGE(0.000001, ABSOLUTE)

/FIXED=Tissue_pH | SSTYPE(3)

/METHOD=ML

/PRINT=G SOLUTION

/RANDOM=INTERCEPT | SUBJECT(PatientID) COVTYPE(VC)

/REPEATED=Brain_region | SUBJECT(PatientID) COVTYPE(UN).

SPSS Syntax 2- To examine the relationship between dependent variable [-ΔCt values of gene of interest] and independent variable [-ΔCt values of potential regulator of gene expression] and adjusting for disease status [Disease_coded] and relevant demographic covariates [demographic data]. Repeat measure of gene expression are for the different brain regions [Brain_regions] in each patient [PatientID].

MIXED [-ΔCt value of gene of interest] BY [Disease_coded] WITH [ΔCt value of potential regulator of gene expression] [demographic data]

/CRITERIA=CIN(95) MXITER(100) MXSTEP(10) SCORING(1) SINGULAR(0.000000000001) HCONVERGE(0, ABSOLUTE) LCONVERGE(0, ABSOLUTE) PCONVERGE(0.000001, ABSOLUTE)
/FIXED=[demographic data] [Disease_coded] [ΔCt value of potential regulator of gene expression] [Disease_coded*[ΔCt value of potential regulator of gene expression | SSTYPE(3)
/METHOD=ML
/PRINT=G SOLUTION
/RANDOM=INTERCEPT | SUBJECT(PatientID) COVTYPE(VC)
/REPEATED=Brain_region | SUBJECT(PatientID) COVTYPE(UN).

SPSS Syntax 3 – General Linear Model (GLM) analyses for the generation of the graphical output to illustrate the relationship between dependent variables [-ΔCt values of gene of interest] for all four brain regions [ACC = anterior cingulate cortex, CER = cerebellum, PUT = putatmen, VC = visual cortex] between controls and disease samples [Disease_coded], and adjusting for relevant covariates [demographic data].

GLM ACC_[-ΔCt value of gene of interest] CER_[-ΔCt value of gene of interest] PUT_[-ΔCt value of gene of interest] VC_[-ΔCt value of gene of interest] BY [Disease_coded] WITH [demographic data]
/WSFACTOR=factor1 4 Polynomial
/METHOD=SSTYPE(3)
/PLOT=PROFILE(Disease_coded*factor1)
/CRITERIA=ALPHA(.05)
/WSDESIGN= factor1

/DESIGN= [Disease_coded] [demographic data]
